# Supplementary figures and images for: HIV infection and cardiovascular disease have both shared and distinct monocyte gene expression features: Women’s Interagency HIV study
Source: PLoS One. 2023 May 19;18(5):e0285926. doi: 10.1371/journal.pone.0285926 (PMC10198505; doi:10.1371/journal.pone.0285926)

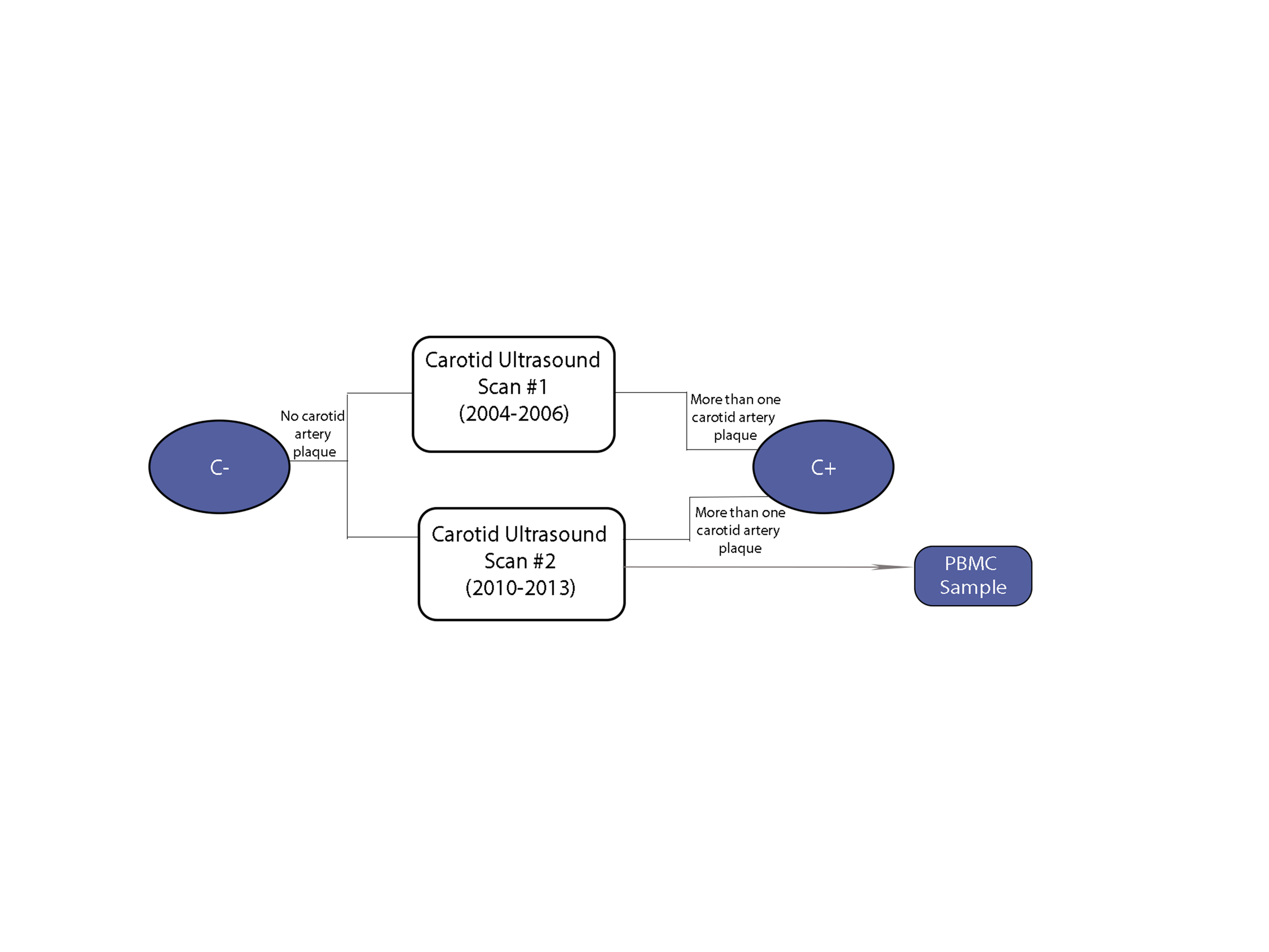

Supplement: S1 Fig — The Women’s Interagency HIV Study (WIHS) was initiated in 1994 and at the time of this study comprised six U.S. sites [17]. Recruitment in the WIHS occurred in two waves (1994–1995, 2001–2002) from HIV primary care clinics, hospital-based programs, community outreach, support groups, and other locations. The WIHS protocol involves semi-annual follow-up visits with detailed examinations, specimen collection, and structured interviews. Incorporated into our definition of cardiovascular disease were data from two waves of B-mode carotid artery ultrasound scans conducted approximately 7 years apart, first during 2004–2006 (Carotid ultrasound substudy scan #1) and again during 2010–2013 (Carotid ultrasound substudy scan #2). For the present report, from both the HIV infected and HIV-uninfected participants of WIHS, we selected groups both with and without subclinical cardiovascular disease (C); the C+ group was defined as those having at least 1 carotid artery plaque assessed at either vascular scan and the C- group was defined as no carotid artery plaque and either of the two scans. Women who used statin drugs for cholesterol lowering were excluded from the C- groups. We withdrew from the repository a sample of peripheral blood mononuclear cells (PBMCs) that had been collected from the semi-annual WIHS core visit that occurred as close as possible in time to the most recent vascular substudy visit. This study is based on transcriptomes of non-classical monocytes isolated from frozen peripheral blood mononuclear cells (PBMCs) obtained from 88 WIHS participants at WIHS Visits 27 to 36 (corresponding to calendar years 2008 to 2012). If samples were not available or had adequate volume, an alternate was selected from an alternate visit before or after the carotid artery ultrasound scan. (TIF) [file pone.0285926.s001.tif]

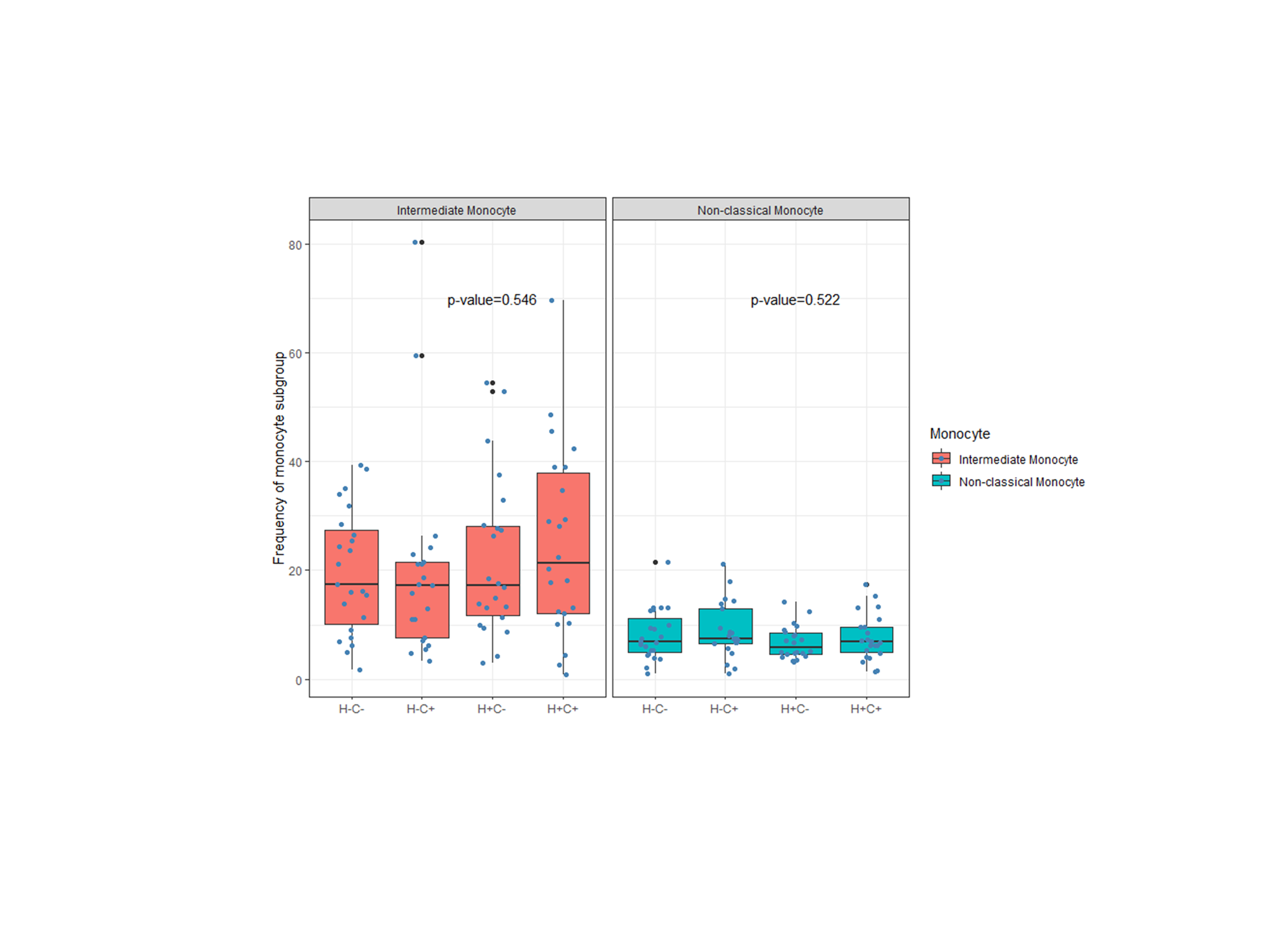

Supplement: S2 Fig — (TIF) [file pone.0285926.s002.tif]

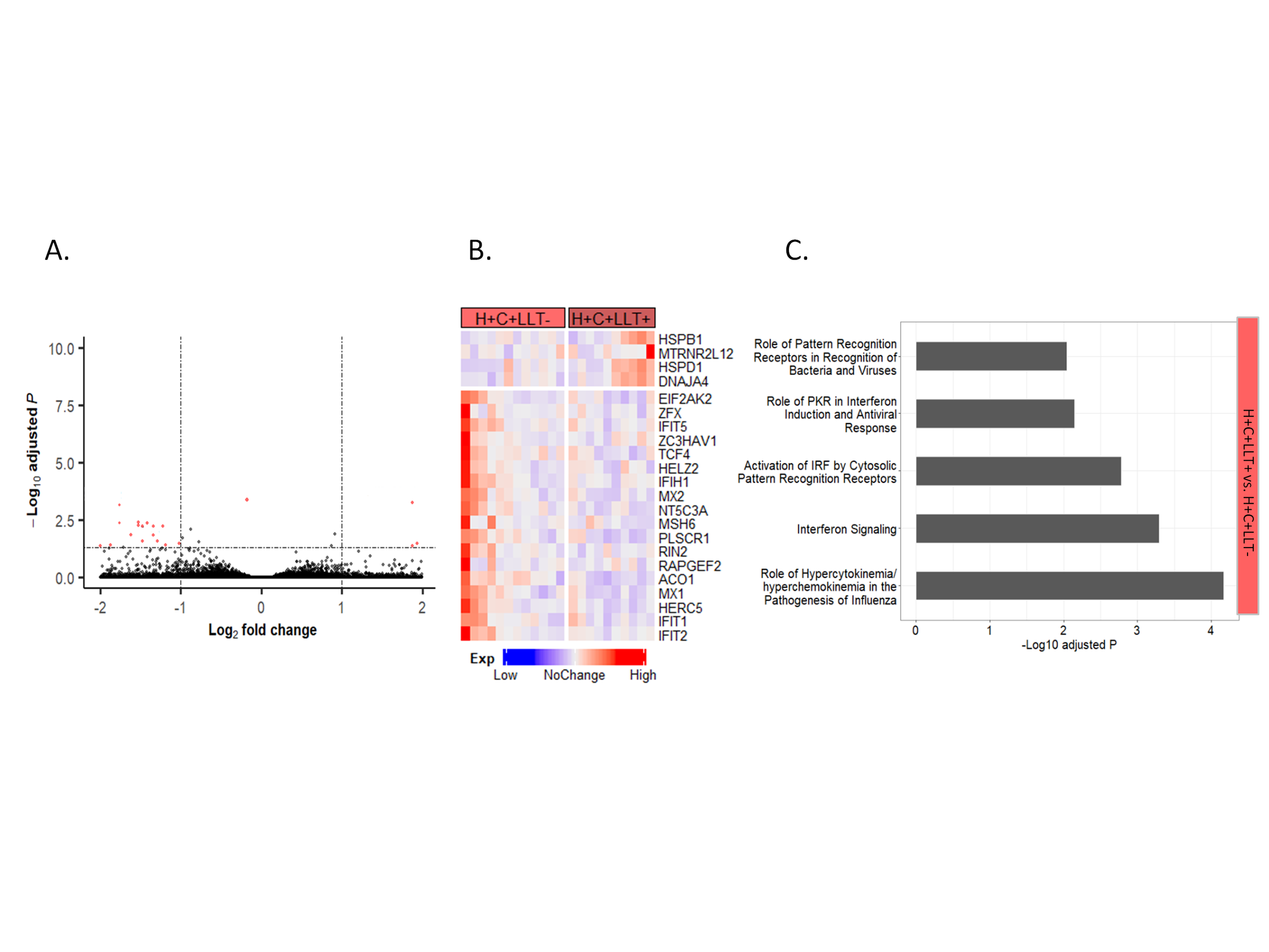

Supplement: S3 Fig — A. Volcano plots for differential gene expression analysis in comparisons of H+C+LLT+ versus H+C+LLT-. Red genes indicate significance by FDR < 0.05 and |log2 fold-change|>1. B. Heatmap showing expression of top up- and down-regulated DEGs found in women with H+C+LLT+ versus H+C+LLT-. C. Top significantly enriched pathways predicted by the fold change of DEGs found in H+C+LLT+ vs. H+C+LLT- comparison, using ingenuity pathway analysis. (TIF) [file pone.0285926.s003.tif]
