# Supplementary material for: HIV infection and cardiovascular disease have both shared and distinct monocyte gene expression features: Women’s Interagency HIV study
Source: PLoS One. 2023 May 19;18(5):e0285926. doi: 10.1371/journal.pone.0285926 (PMC10198505; doi:10.1371/journal.pone.0285926)
Supplement: S1 Table — (DOCX) [file pone.0285926.s004.docx]

**S1 Table** Characteristics of non-classical monocytes study participants, by HIV infection (H) and subclinical cardiovascular disease (C) status.

|  | **H-/C-, N (%), N=23** | **H-/C+, N (%), N=21** | **H+/C-, N (%), N=22** | **H+/C+, N (%), N=22** | *P* |
| --- | --- | --- | --- | --- | --- |
| Matching factors |  |  |  |  |  |
| Age at baseline vascular study visit, years (median, IQR) | 45(40-49.5) | 45(43-52) | 44.5(40-52) | 47(43-52.8) | 0.65 |
| Black race or Hispanic ethnicity | 22 ( 95.7 ) | 20 ( 95.2 ) | 21 ( 95.5 ) | 21 ( 95.5 ) | 0.99 |
| Any history of smoking | 20 ( 87 ) | 18 ( 85.7 ) | 18 ( 81.8 ) | 19 ( 86.4 ) | 0.98 |
| Other demographic and behavior-related characteristics |  |  |  |  |  |
| Completed high school | 14 (63.6) | 12 (57.1) | 13 (59.1) | 12 (54.5) | 0.98 |
| Any current substance use* | 10 ( 43.5 ) | 10 ( 47.6 ) | 10 ( 45.5 ) | 12 ( 54.5 ) | 0.89 |
| Hepatitis C virus infection status | 6 ( 26.1 ) | 8 ( 38.1 ) | 11 ( 50 ) | 13 ( 59.1 ) | 0.13 |
| HIV-related risk factors |  |  |  |  |  |
| Current ART use |  |  |  |  | 1.00 |
| HAART | - | - | 19 ( 86.4 ) | 18 ( 81.8 ) |  |
| ART only | - | - | 1 ( 4.5 ) | 2 ( 9.1 ) |  |
| No ART | - | - | 2 ( 9.1 ) | 2 ( 9.1 ) |  |
| Undetectable HIV-1 RNA level |  | - | 14 ( 63.6 ) | 12 ( 54.5 ) | 0.76 |
| CD4+ count, cells/uL (median, IQR) | - | - | 563.5(387.8-751.2) | 532(265-771.2) | 0.77 |
| CD4/CD8 ratio (median, IQR) | - | - | 0.7(0.3-1.2) | 0.6(0.4-0.9) | 0.50 |
| Cardiometabolic risk factors |  |  |  |  |  |
| Body mass index (median, IQR) | 30.5(27.1-36.6) | 26.9(23.9-31.7) | 29.1(26-34.4) | 28.7(24.2-31.4) | 0.28 |
| Systolic blood pressure, mm Hg (median, IQR) | 125(113-137.5) | 126.5(118.8-143.5) | 122.5(112-131) | 127(109-138.5) | 0.48 |
| History of hypertension | 12 ( 52.2 ) | 15 ( 71.4 ) | 10 ( 45.5 ) | 16 ( 72.7 ) | 0.16 |
| Current hypertensive medication use | 10 ( 43.5 ) | 13 ( 61.9 ) | 10 ( 45.5 ) | 14 ( 63.6 ) | 0.39 |
| Total cholesterol, mg/dL (median, IQR) | 172(148-183) | 177(139.2-204) | 170(149-200) | 194(170.5-217.5) | 0.05 |
| LDL cholesterol, mg/dL (median, IQR) | 96(72-112) | 82(63-120.5) | 93.5(75.2-123.2) | 113(94.5-126) | 0.10 |
| HDL cholesterol, mg/dL (median, IQR) | 55(44-60) | 50.5(43-61.2) | 54(42-59) | 46(40-53) | 0.46 |
| Current use of cholesterol medications | 0 (0) | 5 (23.8) | 0 (0) | 9 (40.9) | <0.01 |
| History of cholesterol medication use | 2 ( 8.7 ) | 12 ( 57.1 ) | 0 (0) | 14 ( 63.6 ) | <0.01 |
| History of diabetes | 6 ( 26.1 ) | 8 ( 38.1 ) | 4 ( 18.2 ) | 4 ( 18.2 ) | 0.39 |
| Creatinine, mg/dL (median, IQR) | 0.8 (0.7-0.9) | 0.9 (0.8-1.0) | 0.9 (0.8-1.0) | 0.9 (0.7-1.2) |  |
| Current aspirin use | 4 ( 17.4 ) | 5 ( 23.8 ) | 4 ( 18.2 ) | 10 ( 45.5 ) | 0.11 |
| Post-menopause | 10 ( 43.5 ) | 7 ( 33.3 ) | 12 ( 54.5 ) | 14 ( 63.6 ) | 0.17 |
| Inflammatory biomarker levels |  |  |  |  |  |
| Galectin-3, ng/mL (median, IQR) | 9.4(8.1-11) | 8.8(7.5-10.4) | 8.9(6-12.6) | 10(8.6-12.5) | 0.68 |
| Galectin-3 binding protein, ng/mL (median, IQR) | 9.2(5-15.8) | 10.5(5.6-12.9) | 15(5.5-28.4) | 14.7(10.5-17.3) | 0.11 |
| sCD163, ng/mL (median, IQR) | 630.2(500.7-1166.9) | 772(464.7-1103.1) | 925.8(701.5-1242.5) | 1013.9(713.6-1411.9) | 0.06 |
| sCD14, ng/mL (median, IQR) | 1681.7(1409.1-1937.3) | 1696.2(1602.1-2043) | 2094.3(1849.3-2240.2) | 2032.9(1810.1-2588.4) | <0.01 |
| IL-6, pg/mL (median, IQR) | 1.9(0.9-2.3) | 1.7(1.1-2.2) | 1.5(1.1-2.3) | 1.3(1.1-2.4) | 0.90 |

*Substance use includes intravenous drug, crack, and cocaine use. ART = antiretroviral therapy, HAART = highly active antiretroviral therapy, IQR = interquartile range.
