# Supplementary material for: HIV infection and cardiovascular disease have both shared and distinct monocyte gene expression features: Women’s Interagency HIV study
Source: PLoS One. 2023 May 19;18(5):e0285926. doi: 10.1371/journal.pone.0285926 (PMC10198505; doi:10.1371/journal.pone.0285926)
Supplement: S2 Table — (DOCX) [file pone.0285926.s005.docx]

**S2 Table** Differentially expressed genes in Intermediate monocytes, comparing groups with cardiovascular disease (C+) with or without HIV infection (H) and lipid lowering treatment (LLT), versus H-C- controls.

| **Comparison** | **Gene Name** | **Log Fold Change** |
| --- | --- | --- |
| H-C+LLT+ vs H-C- | STARD9 | -2.82 |
|  | DZIP3 | -1.58 |
|  | XPNPEP3 | -1.28 |
| H-C+LLT- vs H-C- | PDE7A | -1.04 |
| H+C+LLT+ vs H-C- | HSPD1 | 1.48 |
| H+C+LLT- vs H-C- | IFIT2 | 2.83 |
|  | IL18 | 2.65 |
|  | IFIT3 | 2.99 |
|  | AGRN | 3.37 |
|  | HERC5 | 2.22 |
|  | RAPGEF2 | 1.63 |
|  | IFIH1 | 1.39 |
|  | ZC3HAV1 | 1.41 |
|  | OASL | 2.28 |
|  | IFIT5 | 1.35 |
|  | ZFX | 1.13 |
|  | ISG15 | 2.33 |
|  | MSH6 | 1.33 |
|  | IFIT1 | 2.81 |
|  | TCF4 | 1.19 |
|  | CCL3 | 2.77 |
|  | ISG20 | 1.96 |
|  | MX1 | 1.94 |
|  | PELI1 | 1.90 |
|  | DDX58 | 2.10 |
|  | CCL4L2 | 3.15 |
|  | CMPK2 | 1.81 |
|  | GADD45A | 1.82 |
|  | HELZ2 | 1.30 |
|  | PNPT1 | 1.56 |
|  | DHX58 | 1.42 |
|  | HERC6 | 1.64 |
|  | TLR7 | 1.45 |
|  | RSAD2 | 2.19 |
|  | GRAMD1A | 1.09 |
|  | RIN2 | 1.24 |
|  | USP18 | 2.44 |
|  | TRAF1 | 1.70 |
|  | AC099489.1 | -2.57 |
|  | CLIC2 | 1.26 |
|  | CFB | 3.02 |
|  | RAB3IP | 1.57 |
|  | NT5C3A | 1.06 |
|  | STK26 | 1.19 |
|  | MX2 | 1.00 |
|  | IFI44 | 1.61 |
|  | SPATS2L | 1.59 |
|  | CLIC4 | 1.19 |
|  | PTGS2 | 1.79 |
|  | IL15RA | 1.19 |
|  | LGALS3BP | 1.56 |
|  | OAS2 | 1.22 |
|  | NEXN | 1.69 |
|  | NR1H3 | 1.52 |
|  | CCL3L1 | 1.85 |
|  | XAF1 | 1.31 |
|  | RNF19B | 1.12 |
|  | VSIG4 | 1.74 |
|  | IL7 | 1.97 |
|  | PTPRM | 2.33 |
|  | KCNA3 | 1.38 |
|  | KLHDC7B | 1.58 |
|  | CXCL10 | 2.04 |
|  | SH3D21 | -1.85 |
|  |  |  |
