# Supplementary material for: HIV infection and cardiovascular disease have both shared and distinct monocyte gene expression features: Women’s Interagency HIV study
Source: PLoS One. 2023 May 19;18(5):e0285926. doi: 10.1371/journal.pone.0285926 (PMC10198505; doi:10.1371/journal.pone.0285926)
Supplement: S3 Table — Ingenuity pathway analysis core analysis results for differentially expressed genes in intermediate monocytes related to A. H+C+LLT- and non-classical monocytes related to B. H+C- C. H+C+. (DOCX) [file pone.0285926.s006.docx]

**S3 Table** Ingenuity pathway analysis core analysis results for differentially expressed genes in intermediate monocytes related to **A.** H+C+LLT- and non-classical monocytes related to **B.** H+C- **C.** H+C+.

**A.**

| **Top Ingenuity Canonical Pathways** | **p-value** | **Molecules** | | |
| --- | --- | --- | --- | --- |
| Role of Hypercytokinemia/hyperchemokinemia in the Pathogenesis of Influenza | 7.34E-18 | CCL3,CXCL10,DDX58,IFIT2,IFIT3,IL18,ISG15,ISG20,MX1,OAS2,RSAD2,TLR7 | | |
| Activation of IRF by Cytosolic Pattern Recognition Receptors | 1.10E-06 | DDX58,DHX58,IFIH1,IFIT2,ISG15 | | |
| Systemic Lupus Erythematosus In B Cell Signaling Pathway | 1.11E-06 | IFIH1,IFIT2,IFIT3,IL18,ISG15,ISG20,TLR7,TRAF1 | | |
| Interferon Signaling | 4.12E-06 | IFIT1,IFIT3,ISG15,MX1 | | |
| Role of Pattern Recognition Receptors in Recognition of Bacteria and Viruses | 7.38E-05 | DDX58,IFIH1,IL18,OAS2,TLR7 | | |
| **Top Disease and disorders** | **p-value range** | | **# Molecules** | |
| Antimicrobial Response | 3.03E-03 - 5.20E-22 | | | 21 |
| Inflammatory Response | 3.03E-03 - 5.20E-22 | | | 42 |
| Infectious Diseases | 3.03E-03 - 2.28E-20 | | | 34 |
| Immunological Disease | 3.03E-03 - 5.58E-20 | | | 46 |
| Connective Tissue Disorders | 3.03E-03 - 1.63E-19 | | | 35 |

**B.**

| **Top Ingenuity Canonical Pathways** | | **p-value** | | **Molecules** |
| --- | --- | --- | --- | --- |
| Differential Regulation of Cytokine Production in Intestinal Epithelial Cells by IL-17A and IL-17F | | 1.27E-05 | CCL5,IFNG,IL12A | |
| Role of Hypercytokinemia/hyperchemokinemia in the Pathogenesis of Influenza | | 1.82E-05 | CCL5,IFNG,IFNLR1,IL12A | |
| VDR/RXR Activation | | 4.86E-04 | CCL5,IFNG,IL12A | |
| Communication between Innate and Adaptive Immune Cells | | 4.86E-04 | CCL5,IFNG,IL12A | |
| Differential Regulation of Cytokine Production in Macrophages and T Helper Cells by IL-17A and IL-17F | | 5.83E-04 | CCL5,IL12A | |
| **Top disease and disorders** | **p-value range** | | | **# Molecules** |
| Inflammatory Response | 3.99E-03 - 1.24E-08 | | | 15 |
| Hypersensitivity Response | 3.99E-03 - 1.06E-06 | | | 6 |
| Cancer | 3.99E-03 - 1.17E-05 | | | 11 |
| Oragnismal Injury and Abnormalities | 3.99E-03 - 1.17E-05 | | | 20 |
| Tumor Morphology | 3.99E-03 - 1.17E-05 | | | 6 |

**C.**

| **Top Ingenuity Canonical Pathways** | **p-value** | | **Molecules** | |
| --- | --- | --- | --- | --- |
| CTLA4 Signaling in Cytotoxic T Lymphocytes | 6.04E-04 | | GRAP2,LCK,PPP2R2B | |
| T Cell Exhaustion Signaling Pathway | 9.77E-04 | | BATF,EOMES,LAG3,PPP2R2B,RASD1 | |
| Breast Cancer Regulation by Stathmin1 | 1.34E-03 | | ADGRG3,C5AR2,F2R,GPR171,GPR174,GPR35,HTR2B,PPP2R2B,RASD1 | |
| T Cell Receptor Signaling | 1.35E-03 | | GRAP2,LCK,RASD1 | |
| PKCθ Signaling in T Lymphocytes | 4.67E-03 | | GRAP2,LCK,RASD1 | |
| **Top Disease and disorders** | | **p-value range** | | **# Molecules** |
| Inflammatory Response | | 9.27E-03 - 6.13E-07 | | 30 |
| Immunological Disease | | 9.27E-03 - 3.73E-06 | | 43 |
| Developmental Disorder | | 9.27E-03 - 7.51E-06 | | 26 |
| Endocrine System Disorders | | 9.27E-03 - 7.51E-06 | | 25 |
| Organismal Injury and Abnormalities | | 1.03E-02 - 7.51E-06 | | 75 |
