# Supplementary material for: HIV infection and cardiovascular disease have both shared and distinct monocyte gene expression features: Women’s Interagency HIV study
Source: PLoS One. 2023 May 19;18(5):e0285926. doi: 10.1371/journal.pone.0285926 (PMC10198505; doi:10.1371/journal.pone.0285926)
Supplement: S4 Table — (DOCX) [file pone.0285926.s007.docx]

**S4 Table** Log fold change of differentially expressed genes in non-classical monocytes associated with HIV alone, CVD alone and comorbid HIV/CVD.

| **Gene Name** | **HIV alone (H+C-)** | **CVD alone (H-C+)** | **Comorbid HIV/CVD (H+C+)** |
| --- | --- | --- | --- |
| NUAK1 | 3.335 | 2.251 | 2.370 |
| LAG3 | 4.067 | - | 4.301 |
| THEMIS | 1.738 | - | 3.321 |
| ITGAD | 3.495 | - | 3.235 |
| PLEKHG1 | 3.220 | - | 3.148 |
| ABCD2 | 2.026 | - | 2.919 |
| CCDC141 | 2.657 | - | 2.782 |
| MYO6 | 2.400 | - | 2.743 |
| MCOLN2 | 2.686 | - | 2.579 |
| PTMS | 2.221 | - | 2.542 |
| DAPK2 | 1.416 | - | 2.153 |
| RAB11FIP5 | 2.167 | - | 1.972 |
| JAKMIP1 | 1.571 | - | 1.824 |
| PCNX2 | 1.355 | - | 1.549 |
| ITM2A | 1.394 | - | 1.548 |
| DGKH | 1.350 | - | 1.490 |
| CCL5 | 1.322 | - | 1.479 |
| GRAP2 | 1.038 | - | 1.412 |
| KLRC4 | 1.397 | - | 1.384 |
| TNFRSF9 | 1.338 | - | 1.368 |
| MVB12B | 1.467 | - | 1.347 |
| TTN | 1.391 | - | 1.255 |
| VCAM1 | 5.643 | - | - |
| IFNLR1 | 4.245 | - | - |
| IL12A | 2.509 | - | - |
| KIF19 | 2.501 | - | - |
| JHY | 2.263 | - | - |
| ZBTB32 | 2.019 | - | - |
| ZBED6CL | 1.353 | - | - |
| RAB30 | 1.311 | - | - |
| IFNG | 1.304 | - | - |
| CADM1 | 1.210 | - | - |
| KLRC4-KLRK1 | 1.181 | - | - |
| AKAP5 | 1.029 | - | - |
| C12orf75 | 1.004 | - | - |
| TMEM14C | -1.021 | - | - |
| RETREG1 | -1.401 | - | - |
| GCSAM | - | - | 4.384 |
| STYK1 | - | - | 4.266 |
| GSDME | - | - | 4.169 |
| TPRG1 | - | - | 2.183 |
| HOXC4 | - | - | 2.169 |
| CD2 | - | - | 1.909 |
| GPR171 | - | - | 1.907 |
| EOMES | - | - | 1.896 |
| INPP4B | - | - | 1.878 |
| ENPP5 | - | - | 1.865 |
| KIAA1671 | - | - | 1.821 |
| LANCL3 | - | - | 1.807 |
| TIGIT | - | - | 1.773 |
| TSPAN2 | - | - | 1.761 |
| TTC24 | - | - | 1.685 |
| FRMPD3 | - | - | 1.645 |
| IL32 | - | - | 1.545 |
| C4orf50 | - | - | 1.446 |
| KIF21A | - | - | 1.442 |
| BATF | - | - | 1.436 |
| GZMH | - | - | 1.436 |
| FCRL6 | - | - | 1.413 |
| Z82206.1 | - | - | 1.382 |
| NUGGC | - | - | 1.361 |
| CRTAM | - | - | 1.351 |
| LCK | - | - | 1.269 |
| CMC1 | - | - | 1.169 |
| SVIP | - | - | 1.138 |
| KIAA0040 | - | - | 1.135 |
| F2R | - | - | 1.122 |
| SPATS2L | - | - | 1.119 |
| SLAMF6 | - | - | 1.104 |
| HOPX | - | - | 1.084 |
| ARHGAP11A | - | - | 1.081 |
| PPP2R2B | - | - | 1.072 |
| USP18 | - | - | 1.069 |
| ZNF829 | - | - | 1.056 |
| CD160 | - | - | 1.052 |
| KCNA3 | - | - | 1.043 |
| CD84 | - | - | 1.037 |
| SATB2 | - | - | 1.036 |
| GPR174 | - | - | 1.008 |
| PRR33 | - | - | -1.001 |
| GAS2L3 | - | - | -1.005 |
| BTBD11 | - | - | -1.008 |
| NOCT | - | - | -1.009 |
| PLCXD1 | - | - | -1.010 |
| ZNF469 | - | - | -1.021 |
| IRS2 | - | - | -1.032 |
| BRAT1 | - | - | -1.053 |
| GRASP | - | - | -1.085 |
| HTR2B | - | - | -1.087 |
| ANKRD9 | - | - | -1.123 |
| FAM20C | - | - | -1.138 |
| GSTM2 | - | - | -1.141 |
| ZBTB16 | - | - | -1.156 |
| RASD1 | - | - | -1.174 |
| MSH5 | - | - | -1.195 |
| CSF3R | - | - | -1.267 |
| HBEGF | - | - | -1.296 |
| LMNA | - | - | -1.314 |
| C5AR2 | - | - | -1.320 |
| GPR35 | - | - | -1.327 |
| TRNP1 | - | - | -1.356 |
| ADGRG3 | - | - | -1.437 |
| CEP295NL | - | - | -1.484 |
| ADAMTS1 | - | - | -1.489 |
| KCNN3 | - | - | -1.632 |
| AQP9 | - | -1.347 | - |
