# Supplementary material for: HIV infection and cardiovascular disease have both shared and distinct monocyte gene expression features: Women’s Interagency HIV study
Source: PLoS One. 2023 May 19;18(5):e0285926. doi: 10.1371/journal.pone.0285926 (PMC10198505; doi:10.1371/journal.pone.0285926)
Supplement: S5 Table — (DOCX) [file pone.0285926.s008.docx]

**S5 Table** Differentially expressed genes in non-classical monocytes among women with subclinical cardiovascular disease (C), stratified by HIV infection (H) and lipid-lowering treatment (LLT) status.

| **Comparison** | **Gene name** | **Log fold change** |
| --- | --- | --- |
| H-C+LLT+ vs H-C- | NUAK1 | 2.62 |
|  | B3GAT1 | 2.51 |
|  | NCAPH | 2.45 |
|  | KIAA0391 | 1.80 |
|  | TOGARAM2 | 1.76 |
|  | C13orf46 | 1.46 |
|  | TSPOAP1 | 1.28 |
|  | GNPTAB | 1.06 |
|  | DGKD | 1.03 |
|  | ADCK2 | -1.62 |
|  | TMEM14C | -1.92 |
|  | CPNE2 | -1.94 |
|  | SULT1A1 | -2.18 |
|  | COL24A1 | -2.21 |
|  | GGT1 | -2.34 |
|  | CEACAM1 | -2.63 |
|  | NR6A1 | -3.08 |
|  | IGSF9 | -4.07 |
|  | RASAL2 | -7.22 |
| H-C+LLT- vs H-C- | PLIN2 | -1.02 |
| H+C+LLT+ vs H-C- | MYO6 | 2.51 |
|  | DAPK2 | 2.24 |
|  | MTRNR2L12 | 1.62 |
|  | SLC16A3 | -1.07 |
|  | GRASP | -1.46 |
| H+C+LLT- vs H-C- | PTMS | 2.27 |
|  | CCL4L2 | 2.12 |
|  | CD2 | 1.88 |
|  | CCL4 | 1.85 |
|  | CCL3 | 1.77 |
|  | SPATS2L | 1.76 |
|  | ITM2A | 1.65 |
|  | RSAD2 | 1.57 |
|  | IFIT3 | 1.54 |
|  | IL32 | 1.53 |
|  | ISG15 | 1.46 |
|  | MVB12B | 1.39 |
|  | MX1 | 1.38 |
|  | GRAP2 | 1.33 |
|  | CCL5 | 1.30 |
|  | LCK | 1.30 |
|  | OASL | 1.25 |
|  | CMC1 | 1.13 |
|  | C5AR2 | -1.22 |
|  | PLCXD1 | -1.23 |
|  | CSF3R | -1.33 |
|  | FAM20C | -1.39 |
|  | HIC1 | -1.47 |
|  | CEP295NL | -1.56 |
|  | GPR35 | -1.69 |
