# Supplementary material for: HIV infection and cardiovascular disease have both shared and distinct monocyte gene expression features: Women’s Interagency HIV study
Source: PLoS One. 2023 May 19;18(5):e0285926. doi: 10.1371/journal.pone.0285926 (PMC10198505; doi:10.1371/journal.pone.0285926)
Supplement: S6 Table — (DOCX) [file pone.0285926.s009.docx]

**S6 Table** Gene names of 22 salmon module genes and 70 Magenta module genes in non-classical monocytes.

| **Salmon Module** | **Magenta Module** |  |
| --- | --- | --- |
| LAG3 | SEC14L1 | |
| PLEKHG1 | PLEKHM1 | |
| STYK1 | SLC16A3 |  |
| ABCD2 | RBPJ |  |
| TTN | CXCL16 |  |
| MCOLN2 | PPFIBP2 |  |
| PTMS | CEP295NL |  |
| CCDC141 | STRN4 |  |
| CD2 | C5AR2 |  |
| PCNX2 | PRKCE |  |
| MYO6 | CHST7 |  |
| CRTAM | DNMBP |  |
| ZBTB38 | RREB1 |  |
| RAB11FIP5 | H2AFY |  |
| KCNA3 | ST3GAL5 |  |
| CD84 | H3F3A |  |
| MSC | PABPC4 |  |
| KLRC3 | SNX9 |  |
| SP4 | ELK1 |  |
| HOXC4 | RELT |  |
| SYT11 | MNT |  |
| MPP6 | FAM20C |  |
|  | MED13L |  |
|  | SLC43A2 |  |
|  | TOM1 |  |
|  | NR1D1 |  |
|  | ARHGAP21 |  |
|  | IL10RA |  |
|  | CLASRP |  |
|  | FAM91A1 |  |
|  | IQSEC1 |  |
|  | RCOR1 |  |
|  | QKI |  |
|  | MTMR3 |  |
|  | ADAM9 |  |
|  | PDE8A |  |
|  | SIPA1L1 |  |
|  | PPP1CB |  |
|  | KIF1B |  |
|  | TLE4 |  |
|  | SLC6A6 |  |
|  | PLEKHB2 |  |
|  | ADPGK |  |
|  | CSGALNACT2 |  |
|  | GAS2L3 |  |
|  | PDPK1 |  |
|  | PITPNA |  |
|  | TBC1D12 |  |
|  | SFMBT2 |  |
|  | RHOQ |  |
|  | CLINT1 |  |
|  | SMPDL3A |  |
|  | TENT5A |  |
|  | GAB2 |  |
|  | PDLIM7 |  |
|  | CAMSAP1 |  |
|  | MTMR14 |  |
|  | LPCAT3 |  |
|  | ZEB2 |  |
|  | UHRF1BP1 |  |
|  | AGFG1 |  |
|  | PACSIN2 |  |
|  | TIAM2 |  |
|  | FARP2 |  |
|  | PRKAG2 |  |
|  | ITSN2 |  |
|  | DIRC2 |  |
|  | MTMR10 |  |
|  | ITPRID2 |  |
